# Carbon ion therapy (C12) for high-grade malignant salivary gland tumors (MSGTs) of the head and neck: do non-ACCs profit from dose escalation?

**DOI:** 10.1186/s13014-016-0657-z

**Published:** 2016-07-07

**Authors:** A. D. Jensen, M. Poulakis, V. Vanoni, M. Uhl, N. Chaudhri, P. A. Federspil, K. Freier, J. Krauss, J. Debus

**Affiliations:** Department of Radiation Oncology, University of Heidelberg, INF 400, D-69120 Heidelberg, Germany; Department of Medical Physics, Heidelberg Ion Beam Therapy Center (HIT), INF 450, D-69120 Heidelberg, Germany; Department of Otorhinolaryngology, University of Heidelberg, INF 400, D-69120 Heidelberg, Germany; Department of Dental and Oro-maxillofacial Surgery, University of Heidelberg, INF 400, D-69120 Heidelberg, Germany; Department of Medical Oncology, National Center for Tumor Disease (NCT), INF 460, D-69120 Heidelberg, Germany

## Abstract

**Purpose:**

To evaluate the use of high-dose radiotherapy using carbon ions (C12) on non-adenoid cystic malignant salivary gland tumors (MSGT).

**Patients and methods:**

Between 2009 and 2013, patients with biopsy-proven non-ACC MSGT histologies of the head and neck received a combined regimen of IMRT plus C12 boost. Treatment toxicity (CTC v3), response (RECIST 1.1), control and survival rates were retrospectively analyzed.

**Results:**

40 patients with pathologically confirmed non-ACC MSGT (T4: 45 %; N+: 40 %; gross residual: 58 %; mucoepidermoid carcinoma (MEC): 45 %; adenocarcinoma: 20 %) were treated with a median of 74 GyE (80 Gy BED). Chemoradiation was given in 5 patients with MEC. Grade III acute toxicity was observed in up to 15 % (mucositis, dermatitis, dysphagia), no higher-grade late toxicity occurred to date. At a follow-up of 25.5 months, LC, and PFS at 2 and 3 years are 81.5 % (LC) and 66.8 % (PFS), OS at 2 and 3 years is 83.6 % and 72.8 %. Most frequent site of disease progression was distant metastasis. Histologic subtype correlated with LC and PFS. Resection status (gross vs microscopic disease) had no significant effect on LC, PFS, or OS.

**Conclusion:**

The treatment is well tolerated, no higher grade late effects were observed. Considering the negative pre-selection, LC, PFS and OS are promising. While histology and site of origin significantly influenced control and survival rates, resection status did not, potentially due to the effect of dose escalation.

**Electronic supplementary material:**

The online version of this article (doi:10.1186/s13014-016-0657-z) contains supplementary material, which is available to authorized users.

## Background

Malignant salivary gland tumors (MSGTs) are a heterogenous group of malignancies with more than 20 histological subtypes of different biological and clinical behavior [1].

While adenoid cystic carcinomas (ACC) generally show a very indolent course of disease despite frequent distant metastases [2], this is not the case for other high-grade MSGTs. These may exhibit aggressive local growth patterns as well as early distant disease [3]. Retrospective analyses of larger patient cohorts could establish the use of postoperative radiotherapy and dose-dependence of locoregional control in MSGTs [4–7]. Particle therapy as either neutron or carbon ion therapy has been evaluated in MSGT with promising control rates [8–10]. However, most of these series include a significant proportion of patients with ACC whose natural history of disease and biological behavior is distinctly different from non-ACC histologies. We therefore present our experience with a combined regimen of IMRT plus carbon ion boost in the treatment of non-ACC MSGTs of the head and neck.

## Patients and methods

### Immobilization, target volume definition, and treatment planning

Patients were immobilized with custom-made thermoplastic head masks including shoulder fixation (HeadStep®). Target delineation was based on contrast-enhanced CT and MRI scans. Two target volumes were outlined: CTV1 included the macroscopic tumor and tumor bed, the extended target volume (CTV2) included the CTV1 and typical pathways of spread as well as ispilateral nodal levels (II and III). If the primary tumor was located at or crossing midline, bilateral nodes were included. A 3 mm margin was added to the CTVs to generate PTVs. The PTV margin was reduced at sites were it would extend into critical structures (i.e. optic pathways), no margin was added to organs at risk.

C12 plans were created using Siemens TPS© which incorporates biological plan optimization according to the local effect model (LEM) [11] to account for increased biological effectiveness. Hypofractionation effects are not included in this model; doses need to be converted according to the standard LQ model to yield Gy BED.

IMRT plans were generated either using MRC KonRad© on the Siemens Syngo© platform or tomotherapy©.

### Dose prescription and organs at risk

Both C12 and photon doses were prescribed to the median PTV, which is covered by the 95 % prescription isodose. Normal tissue constraints according to Emami et al. [12] were adhered to. Doses were reduced beyond theses values to as low as reasonably achievable without compromising PTV coverage. Mean dose to at least one parotid gland was kept below 26 Gy.

Patients received a sequential C12 boost in 3 GyE per fraction followed by normofractionated photon IMRT. Most patients received IMRT plus up-front C12 boost (median C12 dose: 23.95 GyE, median IMRT dose: 50 Gy). Two patients underwent C12 only to a total dose of 66 GyE.

### Treatment

C12 treatment was applied using intensity-controlled raster-scanned technique (active beam application) [13] using one to five non-coplanar treatment beams under daily image guidance with orthogonal x-rays and 6 degrees of freedom (DOF) position correction [14]. C12 treatment was routinely available at the Heidelberg Ion Beam Therapy Centre (HIT) in 5 to 6 fractions per week.

IMRT treatments were carried out in step and shoot technique using a 6 MV linear accelerator or tomotherapy unit under regular image guidance with MV cone-beam CT.

### Staging and follow-up

Initial staging included full clinical examination with panendoscopy, local imaging (contrast-enhanced CT/MRI), and chest CT. Patients were followed up 6-8 weeks post completion of treatment, 3 months thereafter and then in 6 monthly intervals with MRI of the head and neck. Yearly chest CTs were recommended to exclude distant disease. In addition, patients were encouraged to regular visits at their referring surgeon incl. full ENT examinations.

### Evaluation

Medical records of all patients treated for pathologically confirmed MSGT between 2009 and 2013 were retrieved and analyzed. Acute and late toxicities were scored according to CTCAE v.3. Tumor staging was performed on initial diagnostic scans to the most recent TNM classification [15], response assessment on follow-up MRI scans according to RECIST 1.1 [16]. Both were carried out by this institution’s MSGT experts.

Time to event data was calculated from radiotherapy treatment start to last follow-up or death according to the Kaplan-Meier method. Loco-regional control (LC) was defined as the absence of further tumor growth following radiotherapy or the absence of further tumor growth following best response of the treated lesion(s). Progression-free survival (PFS) was defined as the absence of locoregional or distant failure or death of any cause. Kaplan-Meier curves were compared using log-rank tests; statistic analyses were performed with the Addinsoft© xlstat life package 2015.

All patients gave written informed consent prior to initiation of treatment, the analysis is in accordance with the current declaration of Helsinki and approved by the institutional review board.

### Ethics and approval

The study was reviewed and approved by the local institutional review board (S-141/2014). The analysis is in accordance with the current declaration of Helsinki.

## Results

Between 11/2009 and 05/2013, 291 patients with MSGTs received carbon ions as part of their primary treatment. 40 patients had pathologically confirmed MSGT other than ACC. Median age was 60 years [range: 35-80 a]. 31 patients underwent surgery prior to radiotherapy, 23 patients had gross residual/inoperable (58 %) and 17 patients had microscopic residual (43 %) disease at RT treatment planning. 7 patients received treatment for locally recurrent MSGT but had not undergone prior radiotherapy. Most common histology was mucoepidermoid carcinoma in 18 patients (45 %) and adenocarcinoma in 8 patients (20 %). Most common sites of origin were parotid gland (50 %) and submandibular gland (13 %). 18 patients (45 %) had T4 stage tumors, 16 patients (40 %) showed nodal metastases.

38 patients received combined radiotherapy with IMRT plus up-front C12 boost to a median dose of 23.95 GyE C12 and 50 Gy IMRT corresponding to a median total dose of 74 GyE and approximately 80 Gy BED. Two patients underwent C12 only to a total dose of 66 GyE for very small primary tumors. With a median CTV1 (C12) of 120 ml and CTV2 (IMRT) of 424 ml, treatment volumes were comparatively extensive (Table 1: patient baseline characteristics).

**Table 1:** Patient baseline characteristics

Patient baseline characteristics				
		All		
		pts	%	
histology	mucoepidermoid carcinoma	18	45	
	adeno carcinoma	8	20	
	acinic cell carcinoma	3	8	
	squamous cell carcinoma	2	5	
	salivary duct carcinoma	2	5	
	NOS	2	5	
	basal cell-adeno carcinoma	2	5	
	adenosquamous carcinoma	1	3	
	myoepithelial carcinoma	1	3	
	basaloid carcinoma	1	3	
stage	T 1	3	8	
	T 2	5	13	
	T 3	13	33	
	T 4a	10	25	
	T 4b	7	18	
	T 4c	1	3	
	unknown	1	3	
	N+	16	40	
	M1	3	8	
treatment for recurrent disease		7	18	
site	parotid gland	20	50	
	submandibular gland	5	13	
	oropharynx	3	8	
	maxilla	2	5	
	palate	2	5	
	lacrimal gland	2	5	
	paranasal sinus	2	5	
	petrous bone	2	5	
	middle ear	1	3	
	nasopharynx	1	3	
age	median in years [range]	60	35-80	
follow-up	all, median in months [range]	25.5	2.5-58.4	
	alive, median in months [range]	27.3	3-58.4	
target volume	CTV1 (boost) in ml	120	34-564	
	CTV2 (extended target volume) in ml	424	105-1538	
dose	C12 in GyE [range]	23.95	17.4-24.4	2 pts: C12 only with 66 GyE
	IMRT in Gy [range]	50	42-56.4	
	total in GyE [range]	74	50.4-74.8	

Five patients with mucoepidermoid carcinoma received combined chemoradiation with cisplatin 40 mg/m sq weekly following interdisciplinary discussion of these specific cases.

Follow-up is 25.5 months [range: 2.5-58.4 months] and 27.3 months [range: 3-58.4 months] for patients alive. 8 patients have deceased due to disease progression.

As expected, mucositis and dermatitis were the most prevalent acute radiation effects. Despite 5 patients undergoing combined chemoradiation, higher-grade mucositis was observed in only 15 % (6 patients). 18 (45 %) patients developed weight loss, however, only 20 % of patients reported dysphagia grade II or III. Trismus was present in 3 patients, in all of those following initial surgery. The most commonly reported late effect was xerostomia grade I (30 %), no higher grade late effects were observed to date. 5 patients developed hearing impairment (13 %) and sensory impairment (13 %), 3 patients rhinitis sicca symptoms (8 %). One patient developed tissue necrosis in the nasopharynx following combined chemoradiation for mucoepidermiod carcinoma of the nasopharynx. Fortunately, this did not result in any late sequelae or cranial nerve impairment (Additional file 1: Table S2: treatment toxicity).

**Fig. 1:**
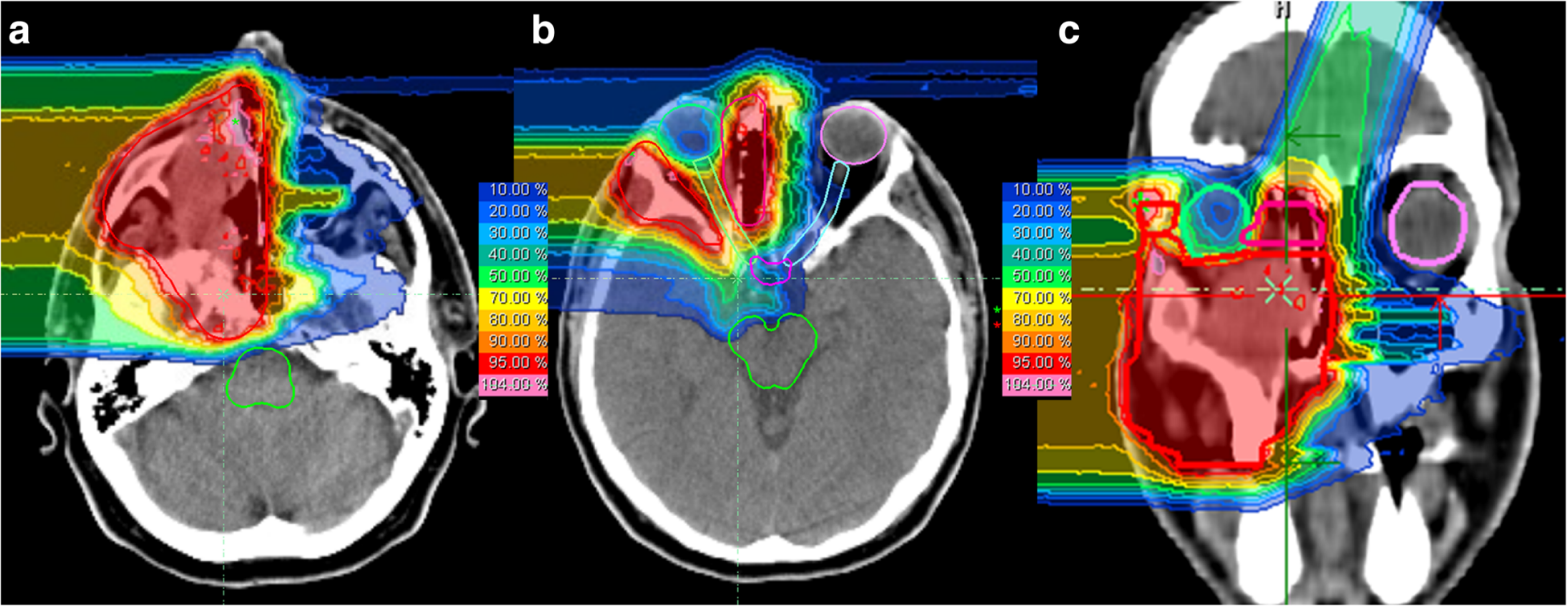
C12 treatment plan of a patient with mucoepidermoid carcinoma of the paranasal sinus undergoing combined IMRT plus C12 boost. C12 plan based on 2 beams, intensity-controlled C12 therapy (ICCT) to 24 GyE in 3 GyE per fraction. **a** and **b**: axial; **c**: coronal distribution

**Fig. 2:**
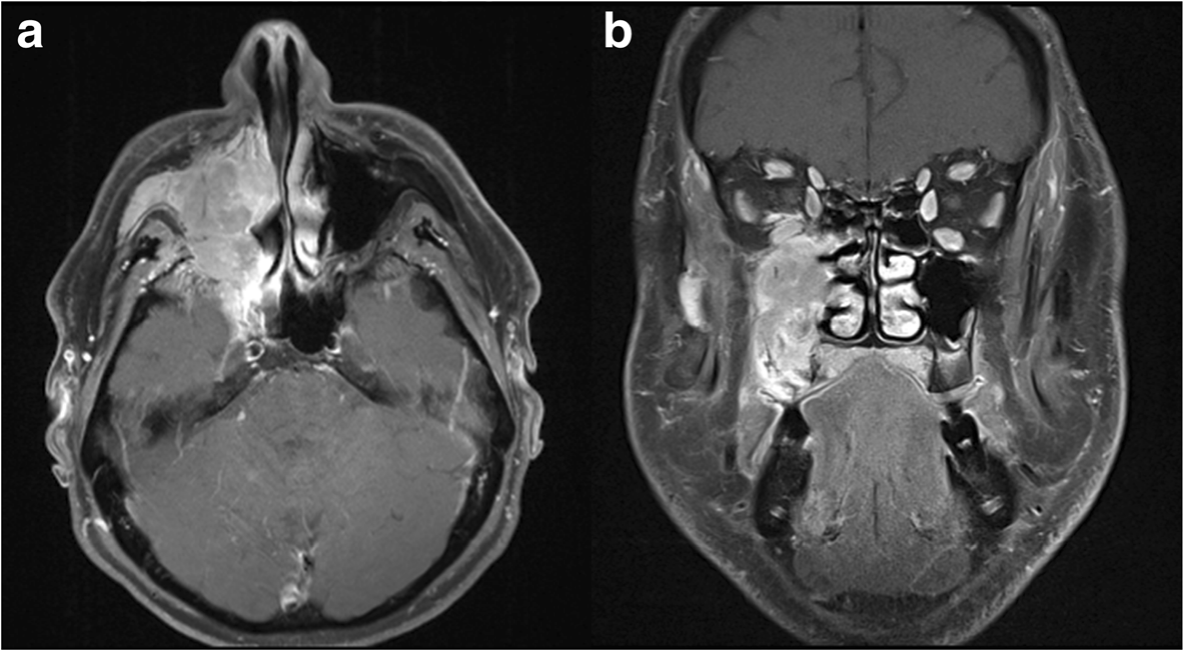
Initial, contrast-enhanced T1-weighted and fat-saturated MRI scan of the patient with mucoepidermoid carcinoma of the paranasal sinus prior to initiation of radiotherapy (**a**: axial; **b**: coronal)

**Fig. 3:**
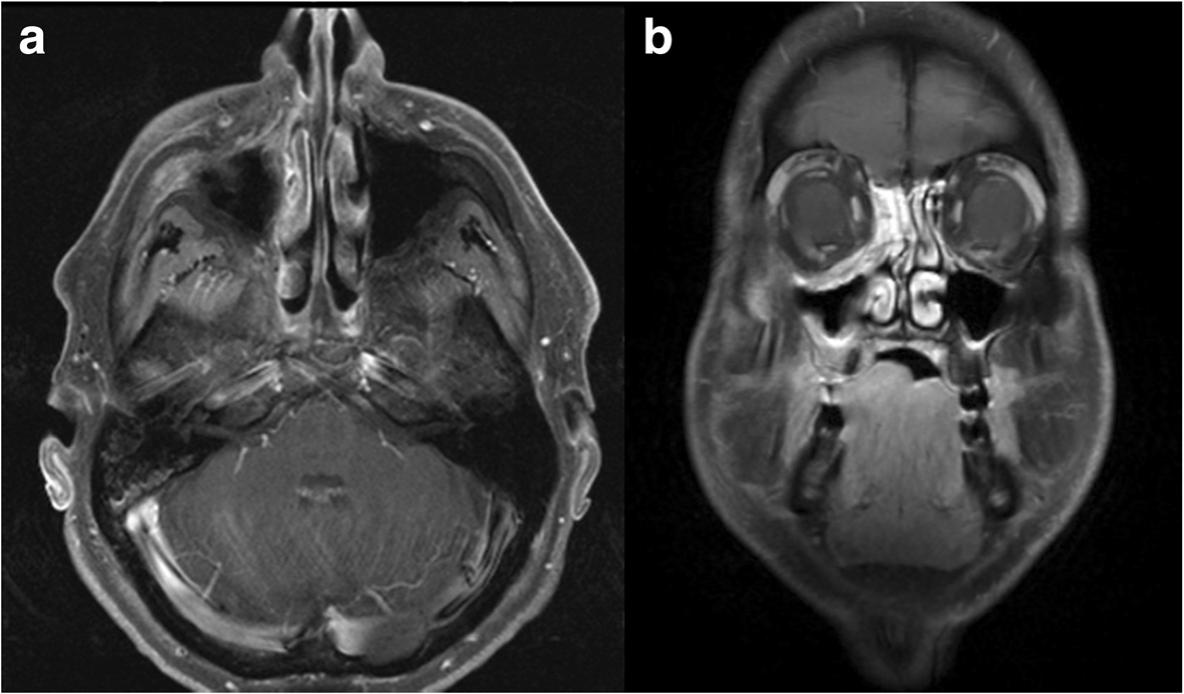
Follow-up contrast-enhanced T1-weighted and fat-saturated MRI scan of the patient with mucoepidermoid carcinoma of the paranasal sinus 6 weeks post completion of radiotherapy (**a**: axial; **b**: coronal)

According to RECIST, 3 patients (8 %) developed complete remissions (CR) 6 weeks post completion of radiotherapy, 10 patients (25 %) partial remissions (PR), 9 patients (23 %) remained stable (SD). In the 16 patients with microscopic residual disease, and in 2 patients deceased prior to first follow-up, response according to RECIST was not assessable. Best response was CR in 7 patients (18 %), PR in 11 patients (28 %), and SD in 4 patients (10 %). Figure 1 shows the dose distribution of C12 plan (24 GyE) for a patient with mucoepidermoid carcinoma of the paranasal sinus, Fig. 2a and b show the initial MRI scan for treatment planning and Fig. 3a and b observed treatment response with a very good partial remission 6 weeks post completion of RT.

**Fig. 4:**
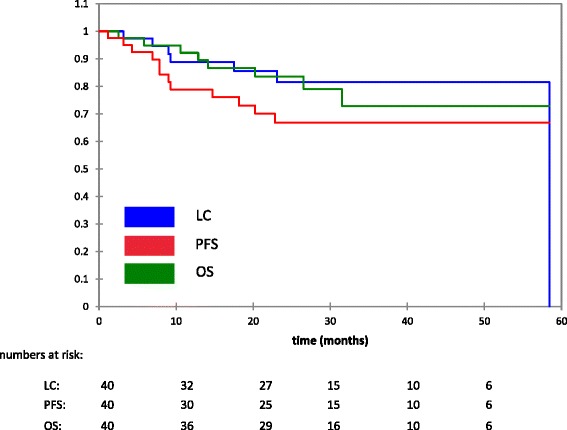
Locoregional control (LC), progression-free survival (PFS), overall survival (OS)

LC, and PFS at 2 and 3 years are 81.5 % (LC) and 66.8 % (PFS), OS at 2 and 3 years is 83.6 % and 72.8 % (Fig. 4).

7 patients developed locoregionally recurrent disease: 3 patients in field, 2 patients at the field edge/gradient where the optic nerve was spared, and 2 patients developed nodal recurrences. 11 patients showed distant disease progression, most commonly as bony (7 pts) and lung (5 pts) metastases, less frequently as soft tissue (2 pts), skin (2 pts), and liver (1 pt) metastases. Further treatment included palliative chemotherapy (6 pts), palliative RT of bone metastases (5 pts), re-irradiation (3 pts), and salvage surgery (1 pt).

**Fig. 5:**
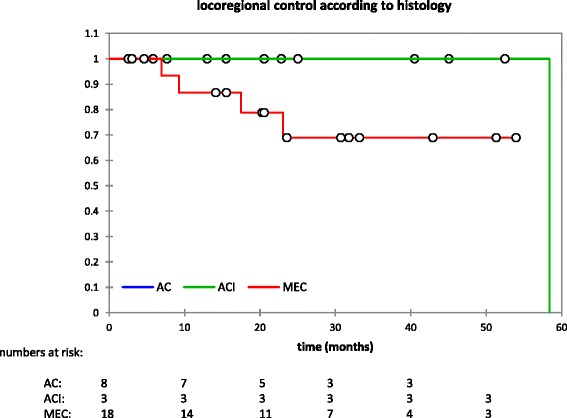
LC according to histologic subtype. AC: adenocarcinoma; ACI: acinic cell carcinoma; MEC: mucoepidermoid carcinoma. Note only the most frequent subtypes are shown to improve visibility

On univariate analysis, LC correlated significantly with histological subtype (p < 0.001). Figure 5 shows LC according to the most common histologies with MEC yielding the least favorable LC. Histology did influence PFS (p = 0.004) (Additional file 2: Figure S1) and distant control (p = 0.003), however, no influence on OS could be detected. Acinic cell carcinoma had the most and adenocarcinoma the least favorable outcome regarding PFS and distant control. While site of origin did not correlate with LC (p = 0.237), it did influence PFS (p = 0.021) (Additional file 3: Figure S2), distant control (p = 0.053), and OS (p = 0.010) with submandibular gland as the least favorable site (Additional file 4: Figure S3). Resection status had no demonstrable effect on PFS, distant control, or OS. Patients with gross residual disease tended to have lower LC, however, this was not statistically significant in this analysis.

## Discussion

While dose escalation using high-LET radiotherapy is seen as beneficial in the treatment of MSGTs and especially ACC of the head and neck [9, 17], the situation is less clear for non-ACC MSGT histologies.

In contrast to ACC, many high-grade non-ACC histologies such as mucoepidermoid carcinoma and adenocarcinoma are characterized by a very aggressive course of disease, frequent nodal and early distant metastases [2, 3]. Adjuvant radiotherapy at doses exceeding 60 Gy is recommended in patients with risk factors such as advanced T-stage, nodal metastases, perineural spread, involved margins, or high-grade histology [2–6].

In our cohort, all patients had at least one of these risk factors: all patients had involved margins and more than 50 % gross residual disease, most patients had advanced tumor stages and 40 % cervical nodal metastases, hence representing a very unfavorable pre-selection [4, 8, 18–21] underrepresented in most other series.

Often, outcome is reported for all MSGTs [5, 7–9] and rarely according to histology [2].

MEC and AC were the most common histologic subtypes in our cohort, also representing two MSGT histologies with the highest potential systemic activity [2, 20–22]. Indeed, most common site of relapse were distant metastases (28 %) which supports findings of Loh, Salgado, Chung and Garden [6, 22–24]. Information on LC in this specific, subset of patients with advanced tumor stages, residual disease and unfavorable histology is rare:

In a large analysis of 207 patients with MSGTs of the major salivary glands treated between 1960 and 2004 at the MD Anderson Cancer Center, LC in node positive patients, advanced T-stages, positive margin or high-grade histology is between 60 and 70 % at 5 years [6]. The MSKCC experience of 98 patients with MSGT of the minor salivary glands treated between 1990 and 2010 yields LC rates of around 80 % at 5 years and significantly inferior results for MECs and adenocarcinoma [22]. LC at 3 years was around 80 % in 54 patients treated with postoperative radiotherapy in the Standford series where advanced T-stage, nodal involvement, and MEC histology was associated with worse outcome [21]. In view of these tumors’ relative radioresistance, neutron radiotherapy has been employed in order to improve control rates. Already in the late 1980s, Griffin and Laramore could show superior LC rates for neutron radiotherapy in the randomized MRC trial albeit with low patient numbers (32 pts) and considerable late toxicity [9, 25]. Stannard et al published their experience in the largest cohort of patients treated with neutrons for MSGTs so far. They found LC rates between 35 % and 50 % for patients with T4 or large tumors, high-grade histology, and nodal metastases [8]. A LC of 72 % at 3 years was reported by Tsuji et al from the NIRS carbon ion center in 26 patients with advanced adenocarcinoma of the head and neck [10].

Despite negative prognostic factors in all our patients, our experience with LC rates of more than 80 % at 2 and 3 years therefore compares favorably with previously published experience.

Inferior results regarding LC and PFS for MEC and adenocarcinoma in our series is supported by other observations and not unexpected [2, 4, 20–22].

Site of origin did influence PFS and OS in our cohort on univariate analysis as reported by other investigators [2, 4, 21, 26]. However, in contrast to all other previously published experience [2, 4, 5, 7, 21], resection status (gross residual tumors vs microscopically involved margins) had no significant influence on LC, which may be an effect of dose escalation to approximately 80 Gy BED.

OS in this cohort is 72.8 % at 3 years and is in line with experience in larger series [7, 8, 24, 27] and the SEER based analysis [20]. It is slightly lower than in the cohort published by Garden [6] which may be due to the high percentage of ACC patients in their series.

As expected, mucositis and dermatitis were the most prevalent acute radiation effects. Despite 5 patients undergoing combined chemoradiation, higher-grade mucositis was observed in only 15 % (6 patients).

No high-grade late toxicity was observed in this cohort so far. Observed toxicity such as hearing impairment and sicca symptoms correlate well with site and extent of treatment field. As described, one patient developed normal tissue necrosis in the high dose area in the nasopharynx following combined chemoradiation. Unfortunately, she still developed an in-field recurrence but does not show any further late toxicity or sequelae. In their retrospective analysis of 98 patients with minor salivary gland malignancies, Salgado et al describe acute and late toxicities including several high-grade late effects (dysphagia, xerostomia, hearing loss) but also 2 out of 98 cases of radiotherapy necrosis, which corresponds to the percentage observed in our cohort [22]. Ten percent of patients developed higher-grade toxicities in the Florida cohort published by Mendenhall et al [7]. The neutron experience reports severe late toxicities in 9 % of patients [8] and significantly higher than in comparable photon treatments [9]. Despite large treatment fields and chemoradiation in 20 % of patients, treatment with IMRT plus carbon ion boost was very well tolerated. Higher-grade acute effects such as mucositis and dysphagia were rare and rates corresponded well to our previously published experience [28, 29]. No temporal lobe changes were observed. In view of the comparatively short follow-up of our patients (25 months), further monitoring is warranted.

No report exists as to observed treatment response of non-ACC of the head and neck so far. Early and best response rates are in line with our previously published experience in MSGT [29]. Still, 3 patients developed in-field recurrences, 2 patients recurrences within the dose gradient towards the optic system. While further dose escalation should be approached with caution, recurrences within the gradient underline the necessity to clearly discuss the issue of sparing specific structures and its potential consequences with the patient.

Chemoradiation in MSGTs is still discussed controversially. There is only limited data on the use of concurrent platin-containing chemoradiation and patient numbers are small [30–34]. It is still unclear whether the addition of chemotherapy to high-dose irradiation does prevent early systemic metastases and even less clear whether the addition of chemotherapy to particle treatment has an added benefit. Once recruitment of the RTOG 1008 trial is completed and results are available [35], we may gain more insight into these issues for photon radiotherapy. In particle therapy however, we do believe these questions should be addressed in a prospective clinical trial.

## Conclusion

Overall acute and late treatment toxicity of the combination of IMRT plus C12 boost remains consistently low, no higher grade late toxicities were observed. LC, PFS and OS are promising, especially considering the negative preselection of patients with advanced disease and gross residual tumors. While histology and site of origin significantly influenced control and survival rates, resection status did not potentially due to the effect of dose escalation, which therefore appears to be beneficial also in non-ACC histologies.

### Previous presentation

Intermediate results were presented as poster at DEGRO meeting in Düsseldorf, in 2014.

### Ethics approval

This study was reviewed and approved by the local institutional review board (S-141/2014).

## Additional files

Additional file 1: Table S1. Treatment-related acute and late toxicity (XLSX 32 kb) Additional file 2: Figure S1. PFS according to histologic subtype. AC: adenocarcinoma; ACI: acinic cell carcinoma; MEC: mucoepidermoid carcinoma. Note only the most frequent subtypes are shown to improve visibility (ODP 16 kb) Additional file 3: Figure S2. PFS according to site of origin (ODP 16 kb) Additional file 4: Figure S3. OS according to site of origin (ODP 16 kb) Additional file 5: Figure S4. LC according to resection status (ODP 17 kb)
